# Exploring facilitators and barriers to medication error reporting among healthcare professionals in Qatar using the theoretical domains framework: A mixed-methods approach

**DOI:** 10.1371/journal.pone.0204987

**Published:** 2018-10-02

**Authors:** Derek Stewart, Binny Thomas, Katie MacLure, Kerry Wilbur, Kyle Wilby, Abdulrouf Pallivalapila, Andrea Dijkstra, Cristin Ryan, Wessam El Kassem, Ahmed Awaisu, James S. McLay, Rajvir Singh, Moza Al Hail

**Affiliations:** 1 School of Pharmacy and Life Sciences, Robert Gordon University, Aberdeen, United Kingdom; 2 Women’s Hospital, Hamad Medical Corporation, Doha, Qatar; 3 College of Pharmacy, Qatar University, Doha, Qatar; 4 Hamad Medical Corporation, Doha, Qatar; 5 School of Pharmacy, Royal College of Surgeons in Ireland, Dublin, Ireland, Dublin; 6 Institute of Medical Sciences, University of Aberdeen, Aberdeen, United Kingdom; University of Birmingham, UNITED KINGDOM

## Abstract

**Background:**

There is a need for theory informed interventions to optimise medication reporting. This study aimed to quantify and explain behavioural determinants relating to error reporting of healthcare professionals in Qatar as a basis of developing interventions to optimise the effectiveness and efficiency of error reporting.

**Methods:**

A sequential explanatory mixed methods design comprising a cross-sectional survey followed by focus groups in Hamad Medical Corporation, Qatar. All doctors, nurses and pharmacists were invited to complete a questionnaire that included items of behavioural determinants derived from the Theoretical Domains Framework (TDF), an integrative framework of 33 theories of behaviour change. Principal component analysis (PCA) was used to identify components, with total component scores computed. Differences in total scores among demographic groupings were tested using Mann-Whitney U test (2 groups) or Kruskal-Wallis (>2 groups). Respondents expressing interest in focus group participation were sampled purposively, and discussions based on survey findings using the TDF to provide further insight to survey findings. Ethical approval was received from Hamad Medical Corporation, Robert Gordon University, and Qatar University.

**Results:**

One thousand, six hundred and four questionnaires were received (67.9% nurses, 13.3% doctors, 12.9% pharmacists). Questionnaire items clustered into six components of: knowledge and skills related to error reporting; feedback and support; action and impact; motivation; effort; and emotions. There were statistically significant higher scores in relation to age (older more positive, p<0.001), experience as a healthcare professional (more experienced most positive apart from those with the highest level of experience, p<0.001), and profession (pharmacists most positive, p<0.05). Fifty-four healthcare professionals from different disciplines participated in the focus groups. Themes mapped to nine of fourteen TDF domains. In terms of emotions, the themes that emerged as barriers to error reporting were: fear and worry on submitting a report; that submitting was likely to lead to further investigation that could impact performance evaluation and career progression; concerns over the impact on working relationships; and the potential lack of confidentiality.

**Conclusions:**

This study has quantified and explained key facilitators and barriers of medication error reporting. Barriers appeared to be largely centred on issues relating to emotions and related beliefs of consequences. Quantitative results demonstrated that while these were issues for all healthcare professionals, those younger and less experienced were most concerned. Qualitative findings highlighted particular concerns relating to these emotional aspects. These results can be used to develop theoretically informed interventions with the aims of improving the effectiveness and efficiency of the medication reporting systems impacting patient safety.

## Introduction

In 1999, the United States (US) Institute of Medicine (IOM) published its seminal report, ‘To Err Is Human: Building a Safer Health System’ [1], that led to greater focus on patient safety practices and research globally. The report called for comprehensive, coordinated efforts by governments, healthcare providers, consumers and others to promote patient safety, setting a minimum goal of 50% reduction in errors by 2004 [1]. While many advances have been made in healthcare practices, an estimated one in ten patients is still being harmed whilst receiving care [2]. In March 2017, the World Health Organization (WHO) published ‘Medication Without Harm, WHO Global Patient Safety Challenge’, to ‘drive a process of change to reduce patient harm generated by unsafe medication practices’ [3, 4]. Medication errors, defined by the National Coordinating Council for Medication Error Reporting and Prevention (NCCMERP) in the US as, ‘any preventable event that may cause or lead to inappropriate medication use or patient harm, while the medication is in the control of the health care professional, patient, or consumer’ [5], are highly prevalent, with associated global costs of US$ 42 billion annually [3, 4]. Interestingly, the goal of the WHO challenge in 2017 is remarkably similar to that of the IOM in 1999, to ‘gain worldwide commitment and action to reduce severe, avoidable medication-related harm by 50% in the next five years, specifically by addressing harm resulting from errors or unsafe practices due to weaknesses in health systems’ [3, 4].

Both the IOM and NCCMERP have strategic aims that highlight the value of effective and efficient medication error reporting systems and practices in reducing error prevalence and severity [1, 5]. Two key goals of NCCMERP are to: stimulate the development and use of medication error reporting systems by healthcare organisations; and to stimulate the review and analysis of error reports leading to the development of recommendations to reduce, and ultimately prevent, errors [5]. There is, however, evidence of widespread and significant under-reporting of medication errors by healthcare professionals [5].

A number of studies have quantified and characterised influences on reporting and potential reasons for under-reporting. Of the surveys published in the literature, most have been conducted in the US, Australia, and the United Kingdom (UK), with findings of barriers towards reporting including: fear of adverse consequences following reporting [6–8, 9, 10]; disagreement over error identification [7–9]; managerial factors [6, 10] lack of knowledge and awareness [11, 12]; lack of feedback [11]; and insufficient training [12]. Whilst there is a dearth of qualitative studies investigating error reporting, there are suggestions that barriers include: time constraints and burden of reporting; selective reporting depending on error severity; anxiety associated with reporting; lack of feedback following reporting; and cultural norms [13–15].

One key limitation of these studies is the lack of consideration of behavioural theories, rendering results to be of less value in development of interventions. The UK Medical Research Council (MRC) framework, ‘Developing and implementing complex interventions’ describes four phases of: intervention development, feasibility/pilot testing, implementation and evaluation [16]. Attention should be paid to theory as part of the development (intervention building) phase. Developers of medication error reporting intervention strategies need to be aware of relevant theories that are likely to result in more effective interventions than empirical approach.

Furthermore, there is a paucity of evidence on the impact of interventions to optimise healthcare professional reporting of medication errors. Evans et al. reported the evaluation of a complex intervention comprising intense education, a range of reporting options, changes in report management and enhanced feedback [17]. A lack of consideration of behavioural theories as part of the intervention development might have contributed to the considerable variation in results of improvement in reporting rates in only certain hospitals. There is therefore a need for research that explains the influences on medication error reporting behaviours in terms of psychological theories.

The Theoretical Domains Framework (TDF) is being used increasingly in healthcare-related research. TDF was developed through expert panel consensus and validation and aims to simplify and integrate the very many behaviour change theories into one framework [18]. The framework derives from 33 psychological theories and 128 theoretical constructs which are organised into 14 overarching domains of behavioural determinants. TDF has been incorporated within intervention developments in the fields of smoking cessation, physical activity, hand hygiene, acute low back pain and schizophrenia [19].

Alqubaisi et al. used TDF in two separate quantitative and qualitative studies of medication error reporting by healthcare professionals in the United Arab Emirates (UAE) [20, 21]. The quantitative study (n = 294) highlighted that the TDF domain of ‘emotional related issues’ appeared to be the dominant barrier to reporting, being common to all health professions [20]. In the qualitative study, key themes that appeared to impact error reporting were: the beliefs of the consequences of reporting; emotions; and issues related to the environmental context. The authors highlighted that these findings may not be generalizable or transferable outwith the study setting and population. There remains a need for further theory informed research on error reporting to confirm these findings. Furthermore, mixed methods research, that will allow quantification of facilitators and barriers to medication error reporting followed by in-depth exploration of any key issues identified, will allow specific targeting of interventions.

The aim of this study was to quantify and explain the behavioural determinants in terms of facilitators and barriers to reporting of medication errors by healthcare professionals in Qatar.

## Methods

### Design

This was a sequential explanatory mixed methods design comprising a quantitative cross-sectional survey followed by qualitative focus groups to gain deeper insight into the survey findings [22, 23].

### Setting

The research was conducted within Hamad Medical Corporation (HMC), the primary provider of secondary and tertiary healthcare in Qatar. At the time of the study, there were eight specialist hospitals employing around 4,000 doctors, 9,000 nurses and 600 pharmacists. HMC policy on medication error reporting has adopted the NCCMERP definition of medication error, with healthcare professionals mandated to report all medication errors and near misses [24]. The reporting system is fully electronic, with all reports being reviewed by the HMC Quality Management Department. Quarterly and annual reports on medication errors and near misses, including action taken are shared with HMC Quality and Patient Safety Committee, and HMC Pharmacy and Therapeutic Committee.

### Cross-sectional survey

The first phase of the research was a cross-sectional survey.

#### Questionnaire development

A draft questionnaire was developed based on published literature on medication error reporting behaviour and associated influences [6–17]. Items on behavioural determinants of error reporting were derived from TDF in the form of the Determinants of Implementation Behavior Questionnaire [25]. This was used in the development of individual questionnaire items, measured using 5-point Likert scales (strongly agree to strongly disagree). In addition, items relating to frequency of submitting medication error reports as well as personal and practice characteristics were included. The draft questionnaire was reviewed for face and content validity by a panel of 10 experts in medication error reporting practice and research in the UK and Qatar.

This was followed by ‘think aloud testing’ with a convenience sample of 20 healthcare professionals in Qatar. This involved each healthcare professional working through the questionnaire individually in the presence of a member of the research team and expressing what they thought in response to each item [26]. Findings resulted in removing several items and rewording others.

The questionnaire was then piloted with a sample of 100 healthcare professionals based in one hospital in Qatar. Test-retest reliability was assessed in all pilot respondents by requesting that the questionnaire be completed again within a two-week interval. A high level of test-retest reliability was achieved with p<0.001 for all Likert statements (Cohen’s weighted kappa).

The findings of all questionnaire pre-testing were incorporated into the final version of the questionnaire which was formatted in Snap Surveys 10 Professional (software for web and email questionnaire design, publication, data entry and analysis). As the common language of care delivery at HMC is English, translation into other languages (e.g. Arabic) was not warranted. A participant information leaflet was developed to provide information on the aim of the study, rationale for inclusion as a participant, potential benefits of participation, estimated time to complete the questionnaire, confidentiality and anonymity. At the end of the questionnaire, respondents were invited to participate in focus group discussions to discuss responses in more detail. Those interested were requested to contact the researchers separately, providing their email address, profession, base hospital, and length of experience as a healthcare professional.

#### Recruitment

All doctors, nurses and pharmacists working within HMC were eligible to participate, with no exclusions. Three hundred and sixty responses were required to give a margin of error of 5% with 95% confidence intervals [27]. Online participation was encouraged via HMC web alerts and promotional posters. In addition, paper-based questionnaires were distributed to all doctors, nurses and pharmacists. Data were collected from mid-January 2016 to mid-April 2016.

#### Data analysis

The survey instrument generated anonymised emails of online submissions that were imported into Snap Surveys before direct export to SPSS version 21.0. Data from paper-based questionnaire were entered manually.

Descriptive statistics were used to describe respondents’ demographics and their responses to other survey item. Five-point Likert scale items relating to TDF behavioural determinants were subjected to principal components analysis (PCA). This is a statistical technique used to reduce a large number of items or variables to a smaller, more manageable number of components [28]. Data suitability for PCA was tested via: determination of the correlation matrix for co-efficient (≥0.3); the Kaiser-Meyer-Olkin measure of sampling adequacy (≥0.6); and Bartlett’s test of sphericity (≤0.05). The number of components was determined via Eigenvalues >1 and inspection of the scree plot. Oblique (Promax) rotation was used to aid the interpretation of the components given that there was reason to assume that selected attitudinal items could be correlated; missing data were excluded pairwise [29]. Where items cross loaded onto more than one component, the item was captured within the component of highest loading. Internal consistencies of the resulting component(s) were tested using Cronbach’s alpha, aiming for >0.60 as desirable for psychometric scales [30]. Total component scores were obtained by assigning scores of 1 (strongly disagree) to 5 (strongly agree) to each of the Likert statement responses (hence treating the ordinal data as interval), with negatively worded items being reverse scored, and generating a summed score for each component.

Differences in total scores among health professions, gender, age and years of experience as a healthcare professional in relation to component scores were tested using Mann-Whitney *U* test (2 groups) or Kruskal-Wallis (>2 groups). P-values ≤0.05 were considered statistically significant.

### Focus groups

To clarify, explore and explain issues identified in the survey phase, a qualitative approach was employed.

#### Sampling and recruitment

Questionnaire respondents who expressed interest in participating in the focus groups were sampled purposively providing a wide range of professions, hospitals and experiences. They were contacted via email and given the option of participating in single or mixed professional focus groups.

#### Topic guide development

The focus group topic guide was developed following analysis of questionnaire findings, with the intention of providing further description and explanation of key TDF behavioural determinants influencing medication error reporting. Case scenarios were also developed to encourage discussion of facilitators and barriers to reporting. The topic guide was reviewed for credibility by the same panel involved in reviewing the questionnaire.

#### Data generation

Focus groups were moderated by two researchers trained in qualitative research methods generally and the conduct of focus groups specifically. The focus groups were held in central locations within HMC, with signed, informed consent obtained from each participant at the outset. When discussing their experiences of medication error reporting, participants were requested to not name any healthcare professionals or patients. Discussions centred on key behavioural determinants that could promote or hinder error reporting (as identified from the analysis of the survey data) and any steps that could be taken to enhance reporting. Discussions were audio-recorded (with permission) and transcribed in full, using a naturalistic approach in which every utterance is transcribed in as much detail as possible. A clear audit trail was maintained with documented details of data gathering to promote dependability [31]. Sampling and recruitment continued to the point of data saturation, at which no new themes were generated from the data analysis [32]. Focus groups were conducted between mid-May to mid-June 2016.

#### Data analysis

Data analysis followed the Framework Approach, a method widely used in applied or policy relevant qualitative research in which the objectives of the investigation are typically set in advance and shaped by the study objectives. The five steps of the approach were: familiarisation; identifying a thematic framework deductively, using TDF domains for coding; indexing; charting; and mapping and interpretation [33]. Two researchers coded each focus group independently, with consensus reached by discussion among the research team.

#### Ethics approval

The study was approved by the ethics committees of Hamad Medical Corporation, Robert Gordon University, and Qatar University.

## Results

### Cross-sectional survey

#### Respondents’ demographics and professional characteristics

One thousand, six hundred and four questionnaires were received, with most (67.9%) from nurses followed by doctors (13.3%) and pharmacists (12.9%), giving an approximate response rate of 11.8% (doctors 7.7%, nurses 12.7%, pharmacists 55.8%). Around three quarters (70.9%) were female, <40 years (76.0%) and almost half (48.1%) with more than 10 years’ experience as healthcare professionals. Respondents had involvement with medicines-related processes as follows: prescribing medicines (15.1%); administering (61.1%); preparation and dispensing (25.9%); and monitoring (42.0%) (Table 1).

**Table 1:** Respondents’ demographic and professional characteristics (N = 1604).

Characteristic	Percentage	Frequency, n
***Current role in the hospital***		
Clinical nurse educator	0.7	12
Clinical pharmacist	2.8	45
Consultant physician	5.4	86
Head/Charge/Specialist nurse	17.1	275
Nurse	50.0	802
Pharmacist	8.9	143
Pharmacy Director/Supervisor/Specialist	1.2	19
Resident Physician	3.5	56
Specialist Physician	4.5	72
Other	5.0	80
Missing	0.9	14
***Age (years)***		
<30	24.2	392
30–39	41.8	670
40–49	21.5	345
50–59	9.5	153
≥60	1.6	25
Missing	1.7	19
***Gender***		
Male	27.6	442
Female	70.9	1137
Missing	1.6	25
***Country of receiving entry-to-practice degree***		
India	42.7	685
Philippines	17.6	283
Egypt	9.3	149
Qatar	9.2	148
Jordan	4.8	77
Other	14.5	231
Missing	1.9	31
***Experience as healthcare professional in hospital (years)***		
<1	1.6	25
1–5	19.1	306
6–10	29.4	471
11–15	21.4	343
16–20	12.0	193
>20	14.7	235
Missing	1.9	31
***In your role do you typically have direct interaction or contact with patients*?**		
Yes	85.6	1373
No	9.0	145
Missing	5.4	86
***Your primary roles in the medicines process are (multiple options could be chosen)***		
Prescribing	15.1	243
Administering	61.1	980
Preparation and Dispensing	25.9	415
Monitoring	42.0	673
Missing	3.1	49

#### Medication error reporting behaviour

Two-thirds of the respondents (66.8%) stated that they had not reported any medication errors in the preceding 12 months (Table 2).

**Table 2:** Number of medication error reports respondents’ recalled submitting in the preceding 12 months (N = 1604).

Number of reports	Percentage (n)
No event reports	66.8 (1072)
1 to 2 event reports	11.7 (187)
3 to 5 event reports	4.7 (76)
6 to 10 event reports	2.1 (33)
11 to 20 event reports	2.2 (36)
21 or more event reports	4.9 (79)
Missing	7.5 (121)

#### Behavioural determinants of medication error reporting

PCA identified eleven components with eigenvalue > 1.0, explaining 68.1% of the variance. As many of the components had only a very small number of items loading, only six components with most items loading were retained (eigenvalues > 1.7), explaining 56.8% of the variance. All six components were found to be internally reliable (Cronbach’s alpha ≥0.7). Responses to items within these six components are given in Tables 3–8. While most components comprised positive responses, the responses to emotions were negative hence inferential analysis was conducted to identify any significant differences among subgroups.

**Table 3:** Component 1, knowledge and skills related to medication error reporting (Cronbach’s alpha 0.938).

Statements	Strongly Agree % (n)	Agree % (n)	Unsure % (n)	Disagree % (n)	Strongly Disagree % (n)	Missing % (n)
I am aware of the definition of a medication error	62.7 (1005)	34.4 (552)	0.6 (9)	0.4 (7)	1.1 (18)	0.8 (13)
I am confident in my ability to recognise all medication errors	47.3 (759)	44.3 (710)	3.7 (60)	2.3 (37)	1.1 (17)	1.3 (21)
I am aware of the difference between a medication error and an adverse drug reaction	65.8 (1056)	30.4 (488)	1.5 (24)	0.6 (9)	0.9 (15)	0.7 (12)
I am aware of the policy relating to medication error reporting in Hamad Medical Corporation (HMC) hospitals	46.0 (738)	42.6 (683)	5.2 (84)	3.4 (55)	1.7 (28)	1.0 (16)
I find the policy straightforward to apply	37.6 (603)	47.4 (760)	11.0 (177)	1.7 (28)	1.1 (17)	1.2 (19)
I am aware of what is expected of me in relation to medication error reporting	36.3 (583)	53.4 (856)	5.1 (82)	2.8 (45)	1.1 (17)	1.3 (21)
I am aware of my responsibility for medication error reporting	40.2 (645)	50.9 (817)	4.6 (74)	1.8 (29)	0.9 (14)	1.6 (25)
I am aware of which medication errors should be reported	38.3 (614)	49.2 (789)	5.7 (92)	3.7 (60)	1.7 (27)	1.4 (22)
I know how to submit a medication error report	41.7 (669)	42.0 (674)	7.7 (123)	5.4 (87)	1.7 (27)	1.5 (24)
I have the ability to report medication errors	39.0 (625)	50.9 (817)	4.7 (76)	2.5 (40)	0.8 (13)	2.1 (33)
I have the necessary experience to report medication errors	32.4 (520)	45.8 (735)	11.3 (182)	7.0 (112)	1.6 (25)	1.9 (30)
I intend to report all medication errors	43.9 (704)	44.3 (711)	7.1 (114)	2.4 (39)	0.8 (13)	1.4 (23)

**Table 4:** Component 2, feedback and support related to medication error reporting (Cronbach’s alpha 0.919).

Statements	Strongly Agree % (n)	Agree % (n)	Unsure % (n)	Disagree % (n)	Strongly Disagree % (n)	Missing % (n)
I receive sufficient encouragement and support from my multidisciplinary team to report medication errors	14.3 (229)	46.8 (751)	23.0 (369)	11.4 (183)	2.6 (42)	1.9 (30)
I receive sufficient encouragement and support from my peers to report medication errors	12.3 (198)	45.2 (725)	22.4 (360)	13.6 (218)	4.4 (71)	2.0 (32)
I receive sufficient encouragement and support from my seniors to report medication errors	16.0 (257)	51.5 (826)	19.5 (312)	8.4 (134)	2.7 (43)	2.0 (32)
I receive sufficient encouragement and support from my organisation to report medication errors	15.8 (253)	50.5 (810)	18.1 (291)	10.5 (169)	3.6 (58)	1.4 (23)
When I submit a medication error report, I am confident that I will receive feedback from the medication error reporting organisation	17.6 (282)	53.6 (859)	14.6 (234)	9.0 (144)	2.8 (45)	2.5 (40)
When I submit a medication error report I am confident that I will receive constructive feedback from the medication error reporting organisation	15.0 (240)	49.9 (801)	19.1 (307)	10.2 (163)	3.3 (53)	2.5 (40)
When I submit a medication error report I am confident that I will receive feedback from the medication error reporting organisation which is appropriate to the severity of the error	16.3 (262)	53.0 (850)	18.1 (290)	7.0 (113)	2.7 (43)	2.9 (46)
When I submit a medication error report I am confident that I will receive feedback from the medication error reporting organisation that focuses on the system and not the individual	15.0 (241)	49.6 (795)	18.5 (296)	10.5 (169)	3.6 (57)	2.9 (46)
I get professional reassurance from each medication error report that I submit	16.8 (270)	40.0 (641)	27.2 (437)	9.7 (155)	3.2 (51)	3.1 (50)
I feel that there is a ‘no blame’ culture in my organisation in relation to medication errors	12.0 (192)	37.1 (595)	22.2 (356)	18.8 (302)	8.4 (134)	1.6 (25)
I feel that there is a positive safety culture in my organisation in relation to medication errors	17.1 (275)	50.4 (809)	17.1 (274)	10.0 (161)	3.8 (61)	1.5 (24)

**Table 5:** Component 3, action and impact following medication error reporting (Cronbach’s alpha 0.856).

Statements	Strongly Agree % (n)	Agree % (n)	Unsure % (n)	Disagree % (n)	Strongly Disagree % (n)	Missing % (n)
I prioritise reporting those medication errors which I consider to be more serious	29.9 (479)	44.8 (719)	9.9 (158)	10.0 (161)	2.4 (39)	3.0 (48)
I believe that each medication error report I submit will be appreciated by my multidisciplinary team	24.8 (398)	44.6 (715)	19.7 (316)	7.1 (114)	2.3 (37)	1.5 (24)
I believe that each medication error report I submit will be appreciated by my peers	21.8 (350)	39.8 (638)	23.8 (381)	10.5 (168)	2.7 (44)	1.4 (23)
I believe that each medication error report I submit will be appreciated by my seniors	23.8 (381)	45.9 (737)	18.5 (296)	7.4 (118)	2.9 (46)	1.6 (26)
I believe that each medication error report I submit can make a significant contribution to my professional practice	48.4 (777)	43.0 (690)	4.7 (76)	1.6 (26)	0.7 (12)	1.4 (23)
I believe that each medication error report I submit can make a significant contribution to the professional practice of others	46.0 (738)	45.4 (728)	4.7 (76)	1.7 (27)	0.9 (14)	1.3 (21)
I believe that each medication error report I submit can make a significant contribution to patient safety	54.6 (876)	39.9 (640)	3.1 (49)	0.5 (8)	0.9 (14)	1.1 (17)
I believe that each medication error report I submit can make a significant contribution to my organisation	47.0 (754)	44.4 (712)	4.4 (71)	1.6 (26)	0.8 (13)	1.7 (28)

**Table 6:** Component 4, motivation related to medication error reporting (Cronbach’s alpha 0.7).

Statements	Strongly Agree % (n)	Agree % (n)	Unsure % (n)		Disagree % (n)	Strongly Disagree % (n)	Missing % (n)
*For me, reporting medication errors is low priority compared to other professional duties	3.9 (63)	14.1 (226)	12.5 (200)		48.8 (782)	18.7 (300)	2.1 (33)
*I am too busy to report medication errors	4.8 (77)	14.2 (228)	15.7 (252)		43.6 (699)	19.3 (309)	2.4 (39)
*I need to be constantly reminded by others to submit a medication error report	3.9 (62)	16.5 (265)	13.4 (215)		47.2 (757)	16.7 (268)	2.3 (37)
Reporting medication errors is something I seldom forget	7.5 (121)	27.2 (437)	14.8 (237)		37.8 (606)	11.0 (176)	1.7 (27)

*, reverse scored

**Table 7:** Component 5, effort related to medication error reporting (Cronbach’s alpha 0.809).

Statements	Strongly Agree % (n)	Agree % (n)	Unsure % (n)	Disagree % (n)	Strongly Disagree % (n)	Missing % (n)
I am likely to report medication errors even if my peers do not	16.8 (269)	60.5 (971)	14.8 (237)	4.5 (72)	1.0 (16)	2.4 (39)
I am likely to report medication errors even if my seniors do not	16.0 (257)	59.0 (947)	15.1 (242)	6.1 (98)	1.0 (16)	2.7 (44)
I am confident that I will report medication errors even if others I work with do not	31.9 (512)	51.4 (824)	10.8 (173)	2.4 (38)	1.2 (20)	2.3 (37)
I believe it is my professional duty to report medication errors which others have made, irrespective of background	34.8 (558)	48.9 (784)	8.9 (143)	4.5 (72)	1.2 (20)	1.7 (27)
For me, reporting medication errors takes very little time	7.8 (125)	41.0 (657)	28.0 (449)	18.5 (296)	2.2 (36)	2.6 (41)
For me, reporting medication errors takes very little effort	7.8 (125)	41.6 (667)	23.5 (377)	21.3 (342)	2.9 (46)	2.9 (47)
I report medication errors even if there is very little time available to do so	30.9 (495)	52.1 (836)	9.7 (155)	4.3 (69)	1.5 (24)	1.6 (25)
Reporting medication errors is compatible with my daily practice	12.0 (192)	50.7 (814)	18.3 (293)	13.2 (212)	3.6 (58)	2.2 (35)
For me, submitting a medication error report is a normal part of my day	24.1 (387)	32.2 (516)	15.7 (252)	17.1 (274)	9.2 (147)	1.7 (28)

**Table 8:** Component 6, emotions related to medication error reporting (Cronbach’s alpha 0.843).

Statements	Strongly Agree % (n)	Agree % (n)	Unsure % (n)	Disagree % (n)	Strongly Disagree % (n)	Missing % (n)
*It is sometimes difficult for me to accept that I have made a medication error	9.0 (145)	31.5 (506)	13.8 (221)	32.9 (527)	11.0 (177)	1.7 (28)
*I feel uncomfortable about submitting a medication error report for an error I have made	6.9 (111)	28.7 (461)	16.0 (257)	35.8 (574)	10.0 (161)	2.5 (40)
*Others I work with will think less of me if I submit a report for a medication error I have made	6.1 (98)	25.1 (402)	23.6 (379)	34.2 (549)	7.7 (124)	3.2 (52)
*I am concerned about any potential reprimand or blame following submission of a medication error report	9.4 (150)	37.5 (602)	20.4 (328)	24.7 (396)	5.4 (86)	2.6 (42)
*I am concerned about the potential impact on my career following submission of a medication error report	10.2 (163)	39.0 (626)	18.8 (302)	23.2 (372)	5.9 (95)	2.9 (46)
*I feel uncomfortable about submitting a medication error report for an error others have made	8.2 (132)	32.1 (515)	19.1 (307)	30.8 (494)	7.7 (124)	2.0 (32)
*I am concerned about the potential consequences of having to include the name of the professional on a medication error report	13.7 (220)	48.3 (774)	14.4 (231)	17.6 (282)	3.4 (54)	2.7 (43)
*Others I work with will think less of me if I submit a report for a medication error they have made	8.3 (133)	31.2 (501)	24.1 (387)	27.0 (433)	6.3 (101)	3.1 (49)
*I am concerned about patient confidentiality by having to include the patient name on a medication error report	15.2 (244)	44.6 (716)	12.0 (193)	21.3 (342)	4.6 (74)	2.2 (35)

*, reverse scored.

#### Component 1, knowledge and skills related to medication error reporting

(Minimum possible scale value = 12 (least positive), maximum = 60 (most positive), midscale = 36)

With a median value of 52 and interquartile ratio (IQR) of 48–58, respondents generally gave highly positive responses, particularly around awareness of the definition of medication error (97.1% agreement) and awareness of the differences between errors and adverse drug reactions (96.2%) (Table 3). The lowest level of agreement was for having the necessary experience to report medication errors (78.2%).

#### Component 2, feedback and support related to medication error reporting

(Minimum possible = 11 (least positive), maximum possible = 55 (most positive), midscale = 33)

With a median value of 41 and IQR of 35–44, respondents generally gave positive responses (Table 4). The most positive responses were in relation to receiving feedback from the medication error reporting organisation (71.2% agreement) and that the feedback would be appropriate to the severity of the error (69.3%) while the lowest level of agreement was around the perception that there was a ‘no blame’ culture in the organisation (49.1%)

#### Component 3, action and impact following medication error reporting

(Minimum possible = 8 (least positive), maximum possible = 40 (most positive), midscale = 24)

With a median value of 32 and IQR of 30–36 (Table 5), respondents generally gave positive responses. The most positive responses were around the belief that each medication error report submitted could make a significant contribution to patient safety (94.5% agreement) and the least positive for the belief that each medication error report submitted would be appreciated by peers (61.6% agreement).

#### Component 4, motivation related to medication error reporting

(Minimum possible = 4 (least positive), maximum possible = 20 (most positive), midscale point = 12)

Respondents generally gave more neutral responses than for the previous three components (median value of 14 and IQR of 12–16, Table 6). While around two thirds of respondents (67.5%) disagreed that reporting medication errors was low priority compared to other professional duties, around one third (34.7%) agreed that reporting medication errors was something that they seldom forgot.

#### Component 5, effort related to medication error reporting

(Minimum possible = 9 (least positive), maximum possible = 45 (most positive), midscale = 27)

With a median value of 34 and IQR of 31–37, respondents generally gave positive responses (Table 7). The majority of respondents (83.7%) agreed that they were confident that they would report medication errors even if others they worked with did not. The lowest level of agreement (48.8%) was for the statement that reporting medication errors took very little effort.

#### Component 6, emotions related to medication error reporting

(Minimum possible = 9 (least positive), maximum possible = 45 (most positive), midscale = 27)

Respondents generally gave the most negative responses for this component (median value of 26 and IQR of 21–30.75, Table 8). These responses were consistent across all items. Almost two thirds of respondents agreed that they were concerned about the potential consequences of having to include the name of the professional on a medication error report (62.0%) and being concerned about patient confidentiality by having to include the patient name on a medication error report (59.8%). Just under half agreed that they were concerned about potential impact on their careers following submission of a medication error report (49.2%) and any potential reprimand or blame following submission of a medication error report (46.9%). There were statistically significant higher scores in relation to age (older more positive, p<0.001 Kruskall-Wallis), experience as a healthcare professional (more experienced most positive apart from those with the highest level of experience, p<0.001 Kruskall-Wallis), and profession (pharmacists most positive, p<0.05 Kruskall-Wallis).

### Focus groups

#### Demographics of participants

Two hundred and ninety-five survey respondents (18.4%) expressed interest in participating in focus groups. Nine focus groups were conducted (duration 45–60 minutes), at which point it was considered that data saturation of themes was achieved. Fifty-four healthcare professionals from different disciplines participated, with just under half (n = 26, 48.1%) being nurses, followed by 18 (33.3%) pharmacists and 10 (18.5%) doctors. Most were highly experienced with only 11 (20.4%) having less than 5 years of experience.

#### Behavioural determinants associated with reporting medication errors

Table 9 gives the key themes that emerged during the focus group discussions. These are mapped to TDF behavioural determinants, identifying each as a facilitator or barrier to medication error reporting. Illustrative quotes are provided for each. Given that the emotions related component generated the most negative scores in the cross-sectional survey, the related subthemes are described in greater detail. (Note, FG–focus group number; D–doctor number; N–nurse number; P–pharmacist number).

**Table 9:** A summary of TDF domains and themes relating to reporting of medication errors, identifying each as a barrier or facilitator.

Domain	Theme	Facilitator	Barrier	Illustrative quotes
Goals	1. Prevention of future medication errors	√		‘*If we report*, *we’ll be aware about this problem and then will try to prevent it in the future*.’ (FG7P1)
	2. Promoting patient safety	√		‘*Yes*, *of course [to report medication errors] for patient safety*. *‘Yeah*, *we must*, *we have to focus on harm of the patient*. *Patient first*.’ (FG7N4)
Knowledge	1. Lack of knowledge in general concerning medication error reporting		√	‘*Yeah*, *but the new staff*, *they don’t know*, *they don’t know about it [medication error reporting]*, *and every two to three months*, *we are bringing new staff and this is not incorporated in the curriculum of the training or the orientation of the staff*.’ (FG2P2)
	2. Lack of knowledge of medication error reporting policy		√	‘*I think the doctors maybe didn’t have orientation about this*. *They don’t know about the policies [medication error reporting] of the HMC*.’ (FG2P1)
	3. Knowledge of medication error reporting processes	√	√	Facilitator—‘*We know how to report a medication error…*’ (FG6P1) Barrier—‘*So the first thing I will tell you very honestly*, *I don’t know how to*. *I don’t know whom to speak to or how to actually report a medication error*.’ (FG6D2)
	4. Expressed need for educational and training	√		‘*Education of staff*, *encouraging the staff and reassuring the staff*.’ (FG4D1)
Skills	1. Possible lack of ability to recognise and report medication errors		√	‘*As I had told before*, *one medication error in my mind is not the same as a medication error in his mind*.’ (FG6P1)
Social/professional role and identity	1. Professional obligation to report medication errors	√	√	Facilitator—‘*Yeah*, *we need to report this medication error*. *If you are… if you are setting aside all this blame-free culture and also*, *you know*, *everyone should come forward to report this error*’ (FG6P1) Barrier—‘*Why should they report also when they don’t feel like reporting*, *feel like acting on it and feel like improving the system*?’ (FG1D1)
	2. Perceived lack of reporting from doctors		√	‘*Based on my experience for monitoring and analysing medication errors since two years ago*, *what is very noticeable is that high reporting*, *it is coming from the pharmacist*, *and there is also a percentage coming from the nurses especially for the administrating error but I never had for doctors*.’ (FG2P1)
Intentions	1. Selectively reporting errors depending on severity		√	‘*If this is going to harm the patient*, *okay in such cases*, *definitely you will report but if it’s something like… like a near miss*, *it never gets reported because we never give it to the patient*.’ (FG6N1)
	2. Reporting for the wrong reason		√	‘*Yeah*, *he’s suffering and he is now collecting any mistake for his colleague*. *He’s not concentrating*. *Now*, *he is just collecting the mistakes for the other people who report*.’ (FG5P2)
Beliefs about consequences	1. Reporting leading to improved practice	√		‘*Well personally*, *yes because it would help in the future*. *Because it would help a lot of nurses to avoid the same error*.’ (FG6N)
	2. Further investigation		√	‘*We bring us here to this committee to discuss the medication errors like imagine someone who has done an error and then he reports*, *and then he’s been called by two to three committees to investigate the errors*. *What he will go back*?’ (FG2P2)
	3. Impacting staff appraisal		√	‘*Does affect the evaluation*. *Do you think that if she does an error and she does administer a wrong medication*, *do you think she will report it*?’ (FG3N1)
	4. Impacting working relationships		√	‘*I will not [report]*, *I mean*, *why would I*? *Because*, *you know*, *I’m thinking about what happened to my friend*. *Isn’t it*? *So even if you tell me a hundred times that’ no you’re going to be safe’*, *I will think… never*.’ (FG4D2)
	5. Lack of confidentiality		√	*‘…and there is no confidentiality*. *That is most important*, *it’s gossiping*. *Everyone knows*. *Those who are not related also know that*.’ (FG4D1&D2)
	6. Lack of feedback		√	‘*So no feedback*, *no appreciation*, *so do you need to take the stress*? *You work*, *do your assigned work*, *go home healthy and peaceful*.’ (FG1D2)
Emotion	1. Fear and worry		√	‘*I think the thing in Qatar is that people are afraid of reporting because they’re afraid*.’ (FG4P2 & D2)
Reinforcement	1. Encouragement to report	√		‘*Yeah*, *if you will ask me I do encourage reporting of cases*. *I will always tell them this is an incident*. *It doesn’t cause you any harm*. *This is a notification*. *This is not a punishment to anybody*.’ (FG3N1)
Environmental context and resources	1. No fair blame culture		√	‘*Actually*, *what I’m thinking about this whole subject is it’s under reported and that’s 100% true*. *And why*, *because I think from my perspective this is a punitive environment that we are living in*.’ (FG6D1)
	2. Time consuming		√	‘*I think it’s more of a headache*. *If you report and then you’re being called for many meetings*. *We already have no time…*’ (FG2D2)

During all focus groups, the issue of reporting medication errors being associated with fear and worry emerged as a key barrier to reporting. For some, it appeared that this fear was real with reporting leading to punishment,

*‘You know people… when people think some error has happened*, *for me they should report openly but they don’t… it won’t happen in [name of hospital] because they are… they are fearful actually*. *People are really… really… punished*.*’* (FG1P1 & FG1D1)

*‘Maybe people are afraid*. *They are afraid if they will be punished or someone or something… They’re afraid*.*’* (FG2N2)

*‘And I think it’s… if you report it*, *there’s a lot of learning*, *but in the thing in… I think the thing in Qatar is that people are afraid of reporting because they’re afraid*.*’* (FG4P2 & FG4D2)

One negative consequence of submitting a medication error report was that there was likely to be further investigation into the error which was a barrier to submitting further reports,

*‘And another thing*, *if you are going to report an error*, *you will not stop there here*. *You will be asked to write a letter*, *you will be asked to for a meeting*, *it doesn’t stop from there*. *Again*, *next time they will ask you give me feedback on this*. *Give me explanation on this*. *So that is the… the… those are the things that compromises when you are reporting an error*.*’* (FG3N)

There was concern that reporting medication errors was likely to affect any evaluation of their performance resulting in less likelihood of reporting medication errors,

*‘Does affect the evaluation*. *Do you think that if she does an error and she does administer a wrong medication*, *do you think she will report it*?*’* (FG3N1)

*‘It will affect [my performance appraisal]… the issue really…they decrease the evaluation*. *So even if you tell me hundred times that ‘no you’re going to be safe’*, *I will think… I will take time before reporting*. *That’s what I’m saying*.’ (FG4P2)

There was also much concern that submitting a medication error report for an error committed by a colleague would damage working relationships. This was expressed by all healthcare professionals at all levels of seniority,

*‘And she said yeah I will report it*, *but she never reported that because we know that it will end up with the… with blame*. *It’s not because I want to protect my colleague*. *It’s because I don’t agree that we should be blamed because this is the system that is provided to us to work in*.*’* (FG3N)

*‘I will not*, *I mean*, *why would I*? *Because*, *you know*, *I’m thinking about what happened to my friend*. *Isn’t it*? *So even if you tell me a hundred times that’ no you’re going to be safe’*, *I will think… never*.*’* (FG4D2)

‘*If anyone is coming to improve you*, *I will like him*. *But if anyone is coming to report against me*, *I will be the enemy of him*.’ (FG9P1)

Many focus group participants perceived that submitted medication error reports were not handled in a confidential manner and that there was potential for the details of the report to be shared with others leading to a lack of trust,

*‘No confidentiality*. *If you did something*, *everybody would know about it*, *but then the people who get to have the authority to report*, *they have to be trusted people*. *They have to have the confidentiality agreement that they will not spread the name*.*’* (FG5P1)

*‘…and there is no confidentiality*. *That is most important*, *it’s gossiping*. *Everyone knows*. *Those who are not related also know that*.*’* (FG4D1 & FG4D2)

The following TDF domains did not feature during focus groups discussions as determinants of medication error reporting: optimism; beliefs about capabilities; memory, attention and decision processes; social influences; and behavioural regulation.

## Discussion

### Statement of key findings

This mixed methods study allowed quantification of issues relating to medication error reporting followed by in-depth exploration of key issues. The cross-sectional survey stage identified that over two thirds of respondents stated that they had not submitted any medication error reports in the preceding 12 months. In PCA, questionnaire items clustered into six components of: knowledge and skills; feedback and support; action and impact; motivation; effort; and emotions. Responses were most negative for the emotions component, with concerns over potential reprimand or blame, impact on reputation and career. Most concern was expressed by younger and less experienced healthcare professionals. On exploring these emotions related issues during qualitative focus groups, several key themes emerged as barriers to reporting: fear and worry; likely investigation follow reporting; impact on evaluation and appraisal processes; that reporting an error committed by a colleague would damage professional relationships; and that reports were not always handled in a confidential manner.

### Strengths and weaknesses

The mixed methods design is a key strength of this study. As defined by Creswell and Clark, this focuses on ‘collecting, analysing and mixing both quantitative and qualitative data in a single study…Its central premise is that the use of quantitative and qualitative approaches, in combination, provides a better understanding of research problems than either approach alone’ [22]. While there are many studies on error reporting behaviours, there is a lack of mixed methods approaches. The use of TDF as a theoretical lens is a further strength, allowing identification of determinants of medication error reporting grounded in accepted psychological behavioural theories [18]. The appropriateness of data suitability for PCA was confirmed through: the ratio of the number of responses to the number of questionnaire items (>5:1); correlation matrix for co-efficients (≥0.3); Kaiser-Meyer-Olkin measure of sampling adequacy (≥0.6); and Bartlett’s test of sphericity (≤0.05) [28, 29].

There are, however, several limitations to the study hence the results and findings should be interpreted cautiously. While an accurate denominator and hence response rate could not be calculated (e.g. those healthcare professionals in management or administration positions may have no roles in medication processes), even when these individuals are excluded, it is likely that the response rate was low, other than for pharmacists. This low response rate may have been due, in part, to the very sensitive nature of medication error reporting. There may also have been issues of other biases, notably social desirability bias, particularly in relation of knowledge based items [22]. Determining component scores involved treating the ordinal Likert scale data as interval and then undertaking analysis using non-parametric approaches. This assumes that the numerical distances between, for example, strongly agree-agree and strongly disagree-disagree are equivalent. While this may be a limitation, it is an approach commonly used in social sciences to allow determination of scale data (median, IQR). Furthermore, as in all self-reported surveys, it was not possible to validate the data. As the study was conducted in secondary care within Qatar, the findings may lack generalisability and transferability to other settings and countries. However, there are similarities in some key findings with other studies in the Middle East and beyond hence it is likely that the issues identified will resonate widely.

### Interpretation of findings

Effective and efficient medication error reporting systems impact patient care through early identification of issues informing safer systems of practice [1, 5]. HMC requires all errors, irrespective of severity, and near misses to be reported [24], hence the finding that less than one third of respondents had submitted any error reports in the last 12 months is likely evidence of significant under-reporting. This situation is not unique to Qatar or indeed the Middle East [7, 11–13], with the consequence that key opportunities to act on reports and improve medication practices are being missed.

Development of effective interventions to improve reporting is based upon the identification of facilitators and barriers and consideration of theories of behaviour change [16]. As noted earlier, one key strength of this study is the incorporation of behavioural theory into the stages of data collection and generation, and analysis. While other quantitative and qualitative studies have identified barriers of reporting [6–15], there has been a lack of attention paid to theoretical underpinning. PCA identified six components, of which the responses to four were positive, one neutral and one negative. In general, respondents perceived that they were knowledgeable and skilled to enable error identification and reporting. Similarly, they viewed that they were provided encouragement and support from the organisation, seniors and peers to report, that their reports would be appreciated at these levels, and that reporting took little time and effort. The component relating to motivation gave more neutral scores, with issues around the priority of error reporting compared to other tasks and being too busy. The scores for the emotions component were much more negative in relation to feelings of discomfort on reporting errors committed by themselves or others, potential reprimand and blame, impact on reputation and career. The finding that younger and less experienced healthcare professionals had statistically significantly lower scores thus being more negative in relation to emotions may provide some evidence for prioritising and targeting these groups to receive intervention. Alqubaisi et al also reported PCA analysis of determinants of error reporting in the United Arab Emirates (UAE), identifying similar issues around emotions [20], which may add to the generalisability of the findings within the Middle East.

On exploring error reporting behaviour in the focus groups, several facilitators emerged, related to the goals of reporting (promoting safety and preventing future errors), knowledge of processes and reinforcement around encouragement to support. The triangulation of data from the quantitative and qualitative elements confirms that knowledge of processes, skills and goals are not key targets for intervention. Most discussion in the focus groups centred on the barriers relating to emotions identified from analysis of questionnaire responses. Fear and worry emerged as a key theme that deterred reporting, with some citing others being ‘punished’ following reporting. There were narratives around intense follow-up investigations that appeared to focus on the individuals involved rather than the system. There was concern that reporting errors could impact future appraisals and career progression as well as negatively affecting professional reputation and relationships.

While several other qualitative studies have identified anxiety being a barrier to reporting [13–15], the mixed methods approach has allowed the specific issues of anxiety to be quantified and explained. Furthermore, the use of TDF enabled mapping off barriers to specific behavioural domains, in this case emotions and related beliefs of consequences. In a study of one-to-one interviews with healthcare professionals in the UAE, Alqubaisi et al [21], identified several recurring themes of fear and impacting career progression and relationships, increasing the likely transferability of the findings. Given that these studies were conducted in the Middle East, it may be that these issues are related to the culture, although issues around emotions have also been identified in the US, Australia and the UK [7–9, 11–15]. Furthermore, many healthcare professionals working in Qatar and the UAE are expatriate.

The findings reported in this study align to the ‘development’ phase of the MRC complex interventions framework. The use of TDF aids the development of behaviour change interventions that are likely to be more effective than those developed without reference to theory [16]. Behaviour change interventions are ‘coordinated sets of activities designed to change specified behaviour patterns’. These are complex and consist of interacting components known as ‘behaviour change techniques’ (BCTs) which are ‘observable and replicable components designed to change behaviour’ [34]. Evidence based BCTs have been mapped to specific TDF domains to facilitate intervention development [34, 35]. Relevant BCTs for those determinants identified during analysis of the quantitative and qualitative data are given in Table 10.

**Table 10:** Mapping of relevant BCTs for optimising medication error reporting and description of BCTs (adapted from [34, 35]).

Relevant behaviour change techniques (BCTs) for domains of beliefs of consequences and emotions	Description of application of these BCTs to medication error reporting interventions
**Beliefs of consequences**	
1. Emotional consequences	Prompt assessment of feelings after reporting a medication error
2. Anticipated regret	Induce or raise awareness of expectations of future regret about not reporting a medication error
3. Social and environmental consequences	Provide information (e.g. written, verbal, visual) about social and environmental consequences of reporting a medication error
4. Comparative imaging of future outcomes	Prompt or advise the imagining and comparing of future outcomes of reporting v not reporting a medication error
5. Vicarious consequences	Prompt observation of the consequences for others when report a medication error
**Emotions**	
1. Reduce negative emotions	Advise on ways of reducing negative emotions to facilitate reporting a medication error (includes ‘stress management’)
2. Emotional consequences	Prompt assessment of feelings after reporting a medication error
3. Social support (emotional)	Advise on, arrange or provide emotional social support (e.g. from colleagues, ‘buddies’ or staff) for reporting a medication error

Interventions based upon these determinants of behaviour are much more complex to develop and implement effectively compared determinants of knowledge and skills that can be effected by education and training [34, 35]. Interventions should be co-developed with representatives of those who will deliver and receive the intervention. Although behaviour change focuses on the individual, commitment will be required at all levels of the organisation from policy makers, leaders and managers to all healthcare professionals and support workers. This is key within any organisation which operates a positive safety culture, defined as being ‘founded on mutual trust, by shared perceptions of the importance of safety, and by confidence in the efficacy of preventive measure’ [36]. It is noteworthy that one qualitative theme identified was the perception of a lack of a fair blame culture within the organisation hence the commitment at all levels of the organisation needs to be very obvious to all.

### Further work

Further research is warranted to focus on the development of the intervention aiming to optimise medication error reporting. Intervention development should be followed by the steps of feasibility and pilot testing, implementation and evaluation in accordance with the MRC framework. The ultimate outcome measures should focus on patient safety, harm and staff beliefs and experiences.

### Conclusion

This study has quantified and explained the key barriers to medication error reporting which appear to be largely centred on issues relating to emotions and related beliefs of consequences. Quantitative results demonstrated that while these were issues for all healthcare professionals, those younger and less experienced were most concerned. Qualitative findings highlighted particular concerns around: fear and worry; likely investigation follow reporting; impact on evaluation and appraisal processes; that reporting an error committed by a colleague would damage professional relationships; and that reports were not always handled in a confidential manner. These results can be used to develop theoretically informed interventions with the aims of improving the effectiveness and efficiency of the medication reporting systems impacting patient safety.
